# Radiologic Diagnosis of Arterial Tortuosity Syndrome in a Pediatric Patient: A Case Report

**DOI:** 10.7759/cureus.99989

**Published:** 2025-12-24

**Authors:** Amina Salah Alkooheji, Neale Nicola Kalis, Gonca Koç, Suad AlAmer, Vimalarani Arulselvam

**Affiliations:** 1 Pathology and Laboratory Medicine, Bahrain Defence Force Royal Medical Services, Military Hospital, Riffa, BHR; 2 Pediatric Cardiology, Mohammed Bin Khalifa Bin Salman Al Khalifa Specialist Cardiac Center, Riffa, BHR; 3 Pediatric Cardiology, Royal College of Surgeons of Ireland-Medical University of Bahrain, Al Sayh Muharraq Governorate, BHR; 4 Radiology, Bahrain Defence Force Royal Medical Services, Military Hospital, Riffa, BHR

**Keywords:** arterial tortuosity syndrome, arterial tortuosity syndrome in a pediatric patient, ats, pediatric radiology, radiologic findings

## Abstract

Arterial tortuosity syndrome (ATS) is a rare autosomal recessive connective tissue disease. It is mainly featured by elongation and tortuosity of the large and medium-sized arteries, along with connective tissue manifestations. In this report, we present a case of a one-year-old male who was diagnosed during the neonatal period after an episode of desaturation at birth, which required admission to the neonatal intensive care unit (NICU) for respiratory support. Imaging revealed tortuosity and kinking of the aortic arch along with left main pulmonary artery stenosis. The family was counselled and offered genetic testing. A multidisciplinary team meeting was held, which concluded with planning for left pulmonary stenosis stenting. The aim of this case is to emphasize the importance of early recognition and diagnosis of rare conditions like ATS through imaging in situations where genetic testing is unavailable.

## Introduction

Arterial tortuosity syndrome (ATS) is a rare hereditary connective tissue disorder that follows an autosomal recessive pattern of inheritance. ATS is marked by torsion, elongation, and twisting of the medium to large arteries, mainly affecting the aorta and pulmonary arteries. This vascular anomaly can lead to further vascular complications such as aneurysms, stenoses, and dissections. In addition to this, patients with arterial tortuosity syndrome may also present with distinctive features of connective tissue involvement, such as skin hyperextensibility, inguinal or diaphragmatic hernias, and joint hypermobility. Other characteristic findings are cardiovascular features like pulmonary artery stenosis, vascular dissection, and vessel tortuosity [1]. Craniofacial features are more noticeable with age; these features include down-slanted palpebral fissures, micrognathia, and blepharophimosis. Skeletal findings like scoliosis, arachnodactyly, or pectus excavatum may further aid in the diagnosis. These clinical signs should prompt further investigation, including imaging and genetic testing [1-3]. Despite advances in understanding the syndrome, the clinical spectrum and natural history of ATS remain incompletely understood, making the management challenging [3]. This case report focuses on a pediatric patient diagnosed with ATS in infancy, detailing the clinical presentation, diagnostic workup, and management approaches that were used in this case. Given the limited reliability of ectoscopic features in infancy, the assessment relied primarily on the characteristic radiological findings, which provided the key elements needed to support the diagnosis and guide evaluation of the relevant differential considerations in circumstances where genetic testing is limited or inaccessible.

## Case presentation

The patient was born early term at 37 weeks and two days of gestation via spontaneous vaginal delivery to healthy consanguineous parents. The amniotic fluid was grade three meconium-stained. He was delivered by a primigravida mother diagnosed with gestational diabetes; she had good glycemic control and no perinatal complications. The patient’s birth weight was 1.95 kilograms, and his APGAR scores were eight and nine at one and five minutes, respectively.

At birth, the patient was found to be desaturating with an oxygen saturation of 86%. He was in respiratory distress upon delivery as he had mild retractions and tachycardia. He was then admitted to the NICU for a septic workup to rule out sepsis and was kept on 0.5 liters of oxygen via nasal cannula, which was discontinued the next day as he was eupnoeic and maintaining more than 95% oxygen saturation on room air. He received ampicillin and gentamicin for five days according to the local hospital guidelines. The antibiotics were discontinued following negative blood culture results. Despite the desaturation, the baby was active and feeding normally. Another episode of desaturation was noted at day eight of life, managed with one liter of oxygen delivered through a nasal cannula. The oxygen therapy was stopped, and he remained clinically stable throughout his admission.

**Figure 1:**
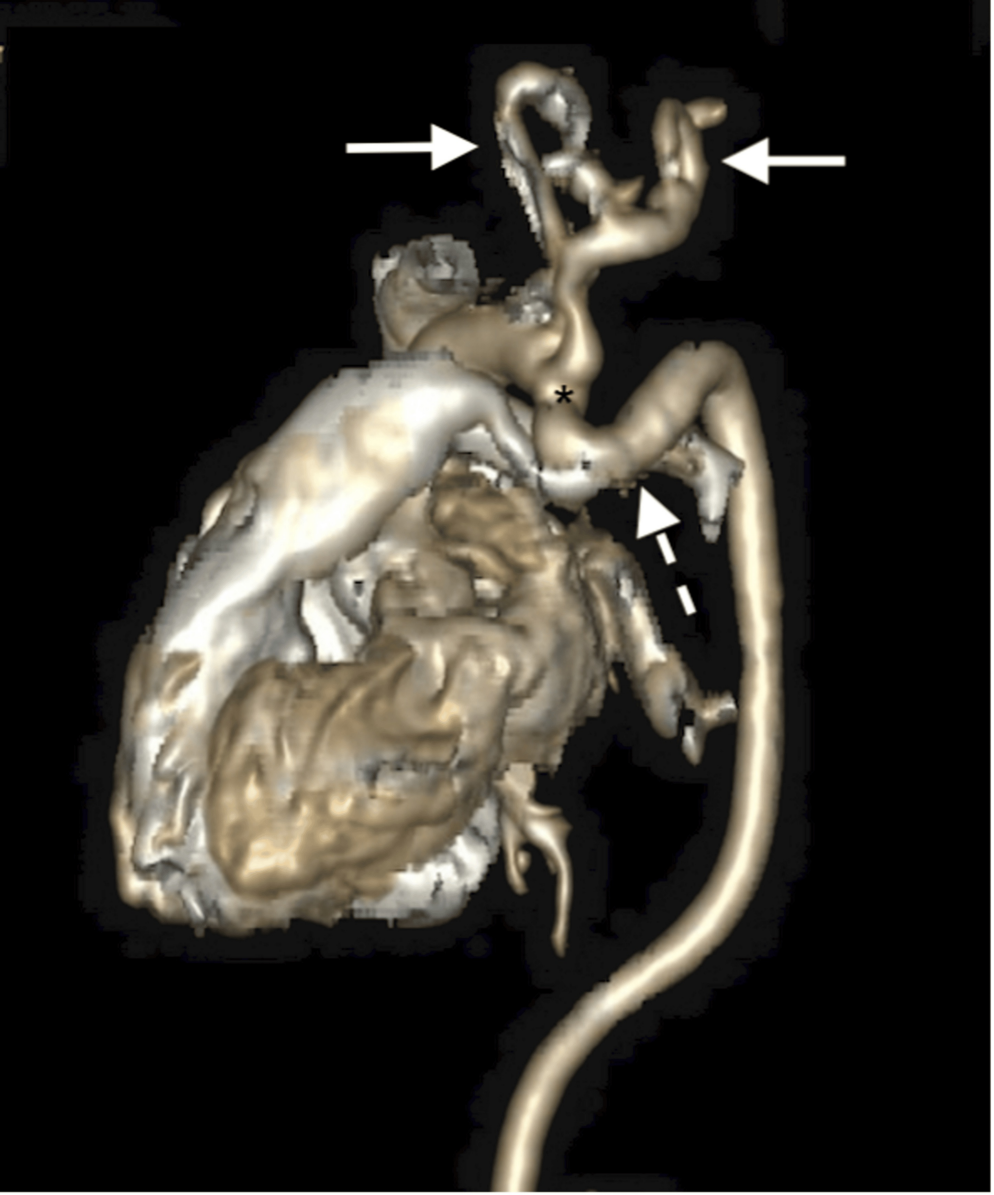
Volume-rendered CT. The image reveals a tortuous aortic arch (dashed white arrow) and its main branches (white arrows). Note the relatively narrow aortic arch just after the origin of the left subclavian artery (asterisk).

On physical examination, the patient was found to have a soft systolic grade III murmur over the left sternal border, which required a transthoracic echocardiographic assessment. The rest of the examination was unremarkable. The echocardiogram was reported as kinking of the aorta, which prompted further evaluation; hence, the patient underwent computed tomography of the chest (CT). The CT findings revealed kinking of the aortic arch, a tortuous course of the proximal descending thoracic aorta, and tortuosity of the neck vessels originating from the aortic arch, with slight narrowing of the aorta distal to the origin of the left subclavian artery, compatible with coarctation as shown in Figure 1. Based on the findings, the diagnosis was suggestive of arterial tortuosity syndrome.

During the hospital stay, the patient showed good weight gain and was feeding well. Before discharge, blood pressure measurements were taken in all four limbs to assess perfusion, on which readings revealed a notable difference between upper and lower limb pressures, with significantly lower blood pressures in the lower limbs: right upper limb: 90/59 mmHg (mean arterial pressure (MAP): 69); right lower limb: 69/37 mmHg (MAP: 49); left upper limb: 91/74 mmHg (MAP: 80); left lower limb: 57/46 mmHg (MAP: 50).

The patient was discharged after 26 days of NICU admission in a vitally and clinically stable condition. He was referred to pediatric cardiology for further evaluation after discharge. As there was no need for immediate intervention, the patient was scheduled for a follow-up imaging appointment to monitor disease progression.

**Figure 2:**
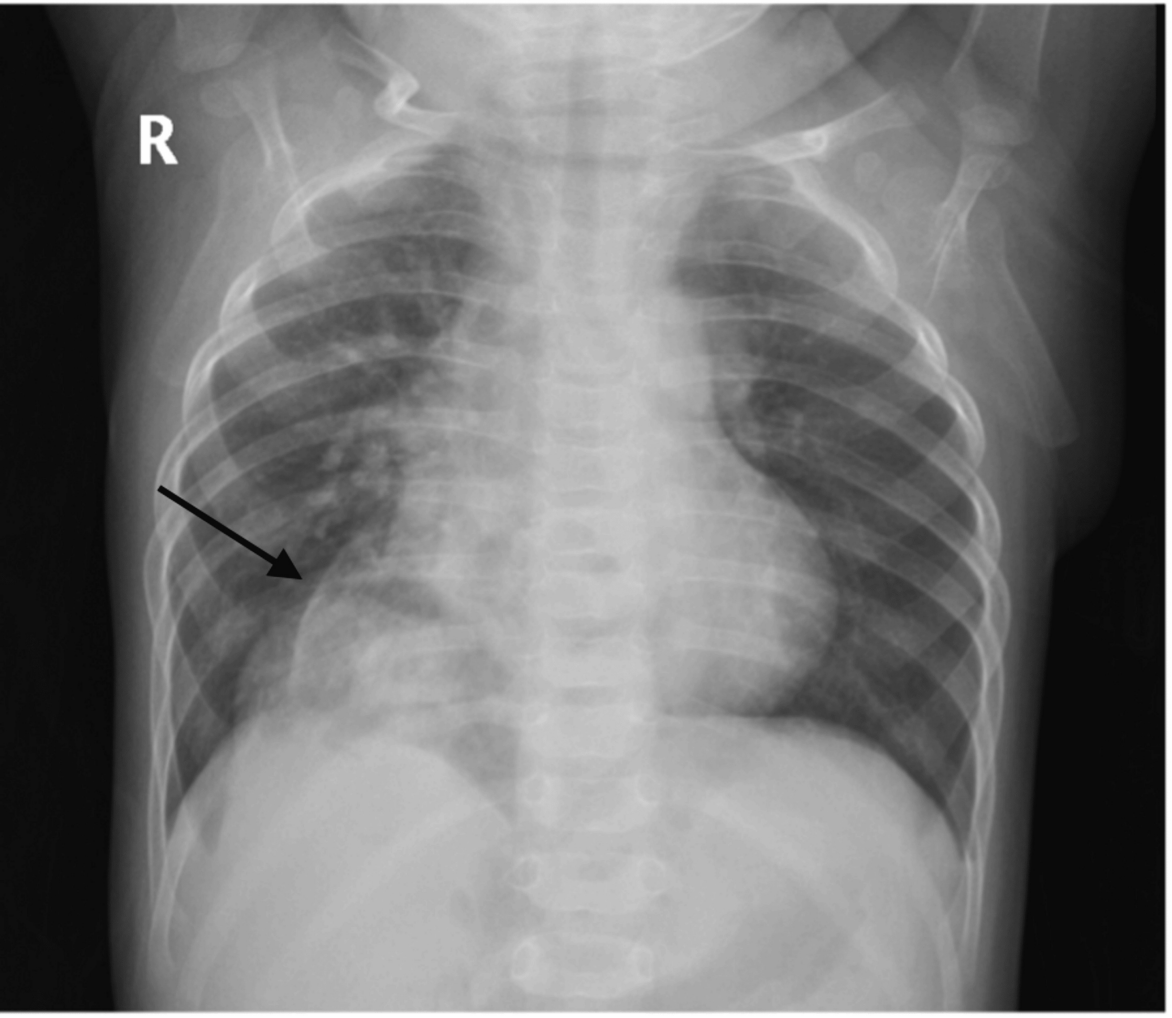
Chest radiograph. The image reveals an abnormal radiolucent area in the right cardio-phrenic angle, containing air-filled bowel loops, consistent with herniated abdominal viscera.

During routine surveillance at eight months of age, a chest X-ray revealed a suspicious finding (Figure 2), prompting referral to pulmonology. A high-resolution CT scan was performed, which incidentally detected a Morgagni hernia that had not been previously identified. The patient was subsequently referred for surgical evaluation. He underwent a successful surgical repair of the hernia, and the postoperative course was uneventful.

**Figure 3:**
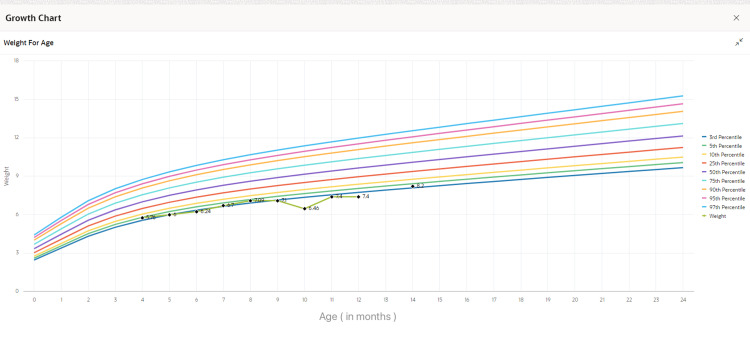
The growth chart shows the patient’s serial weight-for-age measurements plotted over time. The data points indicate faltering growth, with weights consistently tracking below the 10th percentile. This pattern supports a clinical picture of failure to thrive, which may reflect underlying cardiovascular, gastrointestinal, or genetic etiologies.

Between the ages of one month and one year, the patient was noticed to have normal growth parameters; however, his weight gain was lower than expected, as apparent in Figure 3. He had exhibited signs of failure to thrive despite adequate feeding. 

**Figure 4:**
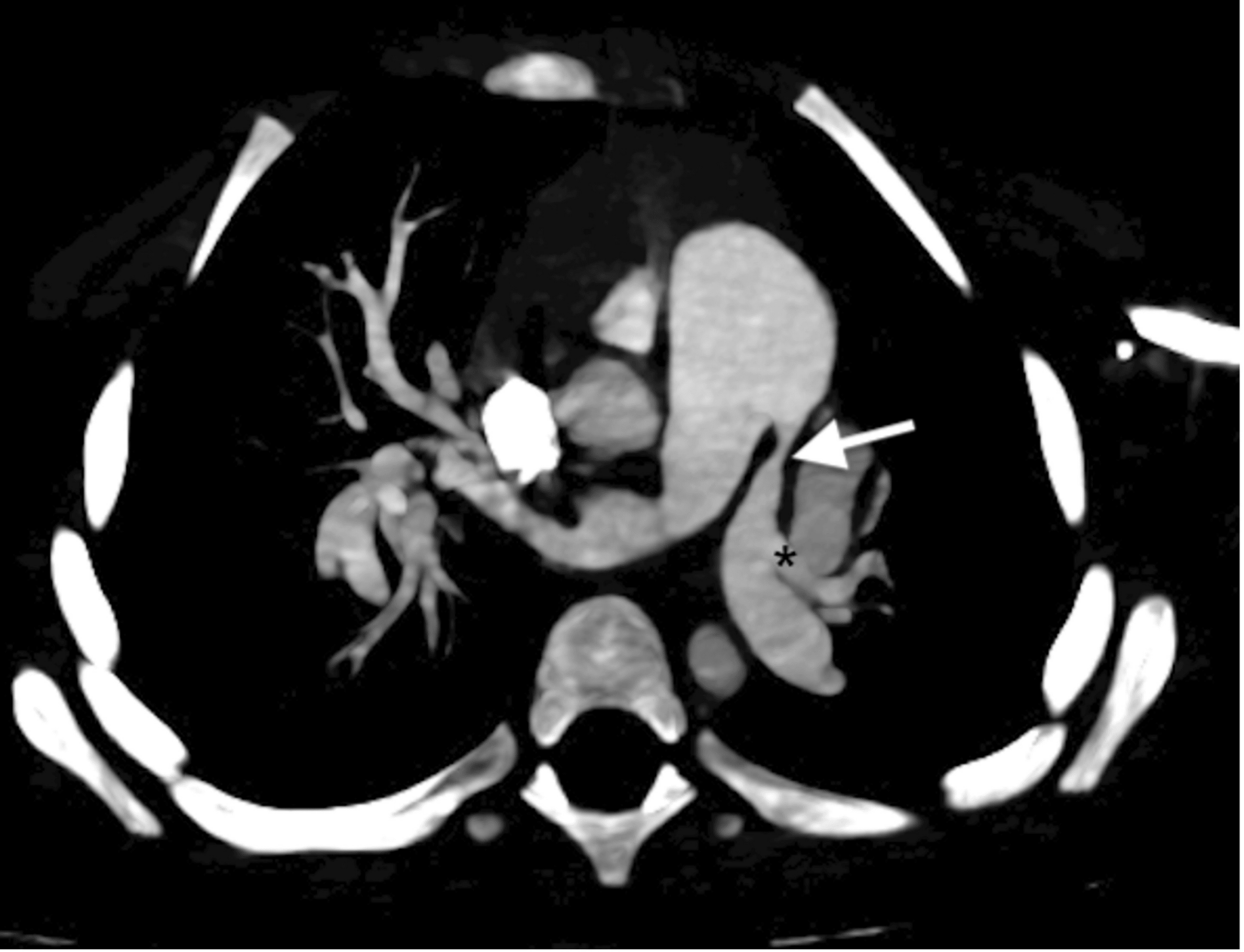
Maximum intensity projection. The CT image shows stenosis of the left main pulmonary artery at its origin (white arrow) and its abnormal second branch (asterisk).

A follow-up CT angiogram was done, which demonstrated marked stenosis of the left pulmonary artery along with an abnormal division of the right main pulmonary artery before the level of the hilum, as shown in Figure 4. Since no other syndromic features were evident, a provisional diagnosis was based solely on imaging.

Currently, at the age of one year and eight months, the patient has remained under close cardiovascular surveillance, including regular follow-up echocardiograms and blood pressure monitoring. His case was discussed in the multidisciplinary meeting, and the final decision was to plan for left pulmonary artery stenting. The patient’s family was counselled for further genetic testing to identify and confirm the causative genetic pathologies. He is under continuous monitoring to prevent any cardiovascular complications and ensure him the best possible quality of life. 

## Discussion

ATS is a rare, autosomal recessive connective tissue disorder that is marked by elongation, torsion, and stenosis of the large and medium-sized vessels, more notably on the aorta and its major branches, along with connective tissue manifestations such as joint hypermobility, joint pain, cutis laxa, and Morgagni hernias. In addition to the connective tissue manifestations, ATS patients might also exhibit craniofacial, skeletal, and ocular findings [1,2,4]. It is classically linked to mutations in the *SLC2A10 *gene, which encodes the *GLUT10* glucose transporter [5,6]. While genetic testing remains the gold standard for confirming the diagnosis, radiological imaging often provides the first crucial clues, especially when the clinical features are subtle or absent. As seen in our case, the diagnosis of ATS was entirely based on vascular imaging, which had highlighted the essential role of radiologic evaluation in early detection and management.

Radiologic diagnosis of ATS

Since standardized diagnostic criteria for ATS are not clearly defined, the diagnostic process generally starts with clinical suspicion, which is supported by imaging modalities such as X-rays, echocardiography, CT angiography (CTA), or magnetic resonance angiography (MRA), and is ultimately confirmed through genetic testing for *SLC2A10* mutations [5]. Imaging plays an essential role in the diagnosis of ATS, with several characteristic signs that might be identifiable on the imaging studies. Some of the key radiological findings include the meandering vessel sign, seen on plain chest X-rays or a coronal CT; it demonstrates tortuous arteries that are extending beyond their usual anatomical boundaries. Another hallmark is the cluster of vessels sign, best appreciated on sagittal or axial CT or magnetic resonance imaging (MRI), which shows the tortuosity of the origins of major arteries, resulting in a dense, cluster-like appearance on cross-sectional imaging. Another important feature is the aortic elongation sign, identified on frontal chest X-rays, characterized by elongation of the aorta, often producing a prominent aortic knuckle, particularly in pediatric patients. In pulmonary imaging, the “V” sign of pulmonary bifurcation is seen in coronal CT or MRI, highlighting early bifurcation of the pulmonary arteries and often associated with narrowing at the origin. Additionally, an inverted "V" sign of pulmonary bifurcation can be seen in axial CT and echocardiography, indicating abnormal narrowing and configuration of the pulmonary arteries [1,7]. 

In our patient, the inverted "V" sign can be appreciated clearly in Figure 4, supporting the suspicion for ATS based on imaging findings. However, this radiologic pattern is not pathognomonic and may also be seen in other heritable connective tissue disorders. Recognition of such imaging features is therefore important to prompt comprehensive cardiovascular evaluation and confirmatory genetic testing.

Differential diagnosis

Several conditions can mimic the presentation of arterial tortuosity syndrome. Due to the overlapping features, it is important to differentiate ATS from other connective tissue diseases, which is essential for accurate diagnosis and management. 

Loeys-Dietz Syndrome (LDS) and ATS both present with arterial tortuosity and aneurysms, but LDS often includes additional features such as hypertelorism, bifid uvula or cleft palate, and generalized arterial aneurysms. It is typically associated with mutations in the *TGFBR1 *or *TGFBR2* genes. Marfan Syndrome is characterized by aortic root dilation, ectopia lentis, and skeletal abnormalities like arachnodactyly, with *FBN1* gene mutations commonly identified. While arterial tortuosity can occur, it is less pronounced than in ATS. Ehlers-Danlos Syndrome (EDS), particularly the vascular subtype, can closely mimic ATS, presenting with translucent skin, easy bruising, and fragility of arteries, intestines, or uterus, and is associated with *COL3A1* mutations. Cutis Laxa Syndromes feature loose, inelastic skin and may also include vascular anomalies such as arterial tortuosity, with differentiation reliant on clinical and genetic evaluation. Other conditions, such as homocystinuria and connective tissue disorders linked to arterial tortuosity, should also be considered in the differential diagnosis [2,8].

In our case, the patient presented with notable arterial tortuosity on imaging, and the clinical features were suggestive of ATS. Although genetic testing for the SLC2A10 mutations was not performed, the combination of radiologic and clinical features strongly supported the diagnosis.

Clinical relevance of imaging-based diagnosis

Imaging plays a pivotal role in the timely and accurate diagnosis of arterial tortuosity syndrome, particularly given the phenotypic overlap with other connective tissue disorders mentioned above. Multimodal imagining, like sonography, CTA, or MRA, is critical not only for identifying characteristic features like arterial elongation and tortuosity, but also for recognizing potentially fatal consequences, including aneurysms, stenoses, and dissections. Serial imaging plays a vital role in the ongoing evaluation of the disease. It allows for monitoring and tracking the vascular progression and guides the therapeutic strategies, including initiating medical management or proceeding with a surgical intervention. It may also act as a diagnostic tool in cases where genetic testing is inaccessible, hence it highlights the importance of imaging in the comprehensive management of ATS [5,7].

Prognosis and long-term follow-up

The prognosis of arterial tortuosity syndrome varies, as it depends on the severity of the vascular involvement and the presence of extravascular complications. Even though some patients remain asymptomatic or have mild symptoms, others may experience severe or progressive vascular complications that might eventually lead to surgical intervention [8]. Given the progressive nature of ATS along with its life-threatening vascular complications, it is essential to have a long-term structured surveillance plan. Regular cardiovascular follow-ups are recommended, including echocardiography every three months up to the age of five. Additionally, annual head-to-toe MRA or CT, starting at diagnosis, is advised to monitor for any evolving vascular pathologies. Moreover, blood pressure monitoring should be measured at each clinic visit to identify and manage hypertension, if present. Orthodontic assessment, spinal radiographs, and ophthalmic evaluations for refractive errors and keratoconus are advised, especially during periods of growth. Pulmonary evaluations may also be recommended if respiratory symptoms are present. A multidisciplinary care approach involving cardiology, genetics, radiology, orthopaedics, ophthalmology, and dentistry is crucial for optimizing long-term outcomes in children with ATS [2].

Limitations and considerations

Arterial tortuosity syndrome is a rare autosomal recessive disease. Due to its rarity, the diagnostic and management guidelines are limited, and the clinical management largely depends on the expertise and judgment of specialists [3]. Most of the arterial tortuosity syndrome data are derived from small case series and individual case reports, which restricts the ability to generalize the findings and establish evidence-based protocols. The exact prevalence of ATS remains unknown, though it is considered a rare syndrome occurring in fewer than 1 in 1,000,000 live births. Nonetheless, some researchers propose that ATS may be underdiagnosed and thus more common than currently reported [2,9]. Given that only around 100 cases of ATS have been reported to date, this disease remains rare and poses significant challenges in both the diagnosis and management [10,11].

## Conclusions

This case highlights the critical role of imaging in early recognition of arterial tortuosity syndrome, particularly when genetic confirmation is not immediately available. Although definitive diagnosis will require molecular testing, the distinct radiologic pattern observed here allows for a provisional diagnosis, initiation of surveillance, and coordination of care. As awareness of ATS increases, more children may benefit from early, non-invasive identification and management, potentially improving long-term outcomes in this rare but serious condition.
